# The risk and added values of the atherosclerotic cardiovascular risk enhancers on prediction of cardiovascular events: Tehran lipid and glucose study

**DOI:** 10.1186/s12967-020-02686-1

**Published:** 2021-01-06

**Authors:** Farzad Hadaegh, Samaneh Asgari, Fatemeh Moosaie, Meysam Orangi, Farzaneh Sarvghadi, Davood Khalili, Fereidoun Azizi

**Affiliations:** 1 Prevention of Metabolic Disorders Research Center, Research Institute for Endocrine Sciences, Shahid Beheshti University of Medical Sciences, Tehran, Islamic Republic of Iran; 2 Tehran University of Medical Sciences, Tehran, Iran; 3 Endocrine Research Center, Research Institute for Endocrine Sciences, Shahid Beheshti University of Medical Sciences, Tehran, Iran; 4 Department of Biostatistics and Epidemiology, Research Institute for Endocrine Sciences, Shahid Beheshti University of Medical Sciences, Tehran, Iran

**Keywords:** Atherosclerotic cardiovascular disease, Risk enhancing factors, American College of Cardiology, The American Heart Association

## Abstract

**Background:**

In 2013 American College of Cardiology and the American Heart Association released a guideline on the management of atherosclerotic cardiovascular disease (ASCVD) including a composite of death from CVD, non-fatal myocardial infarction, or non-fatal stroke (hard CVD). This guideline recommended a risk score that was calculated using pooled cohort equations (ASCVD-PCE). The guideline was updated in 2018/2019 and further risk discussion was suggested for deciding whether to continue or initiate statin therapy among non-diabetic individuals with ASCVD-PCE score ranged 5–20%. They recommended a risk discussion with considering risk enhancing factors (ASCVD-REFs) including family history of premature CVD, chronic kidney disease, triglycerides ≥ 175 mg/dl, low-density lipoprotein cholesterol (LDL-C) ≥ 160 mg/dl, metabolic syndrome (Mets), and for women premature menopause, and hypertensive disorders of pregnancy (HDP). In the current study, we aimed to examine the predictability of recommended ASCVD-REFs on incident hard CVD in non-diabetic individuals with LDL-C 70-189 mg/dl, with ASCVD-PCE risk 5–20% during 10 and 15-year follow-up.

**Methods:**

**A**mong a total of 3546 non-diabetic individuals aged 40-75 years, after excluding those with ASCVD-PCE score < 5% and ≥ 20% (n = 2342), 1204 individuals (women = 332) were included. The univariable and multivariable (further adjusted for ASCVD-PCE) Cox regression analysis were used to evaluate the association of each potential ASCVD-REFs with hard CVD. Additionnaly, the role of different components of Mets and a history of gestational diabetes (GDM)/macrosomia was also examined. The predictive ability of each significant ASCVD-REFs, then was evaluated by the discrimination accuracy and risk reclassification index.

**Results:**

During the 10-year follow-up, 73 hard CVD events occurred. Although in univariable analysis, high blood pressure (BP) component of Mets, GDM/macrosomia, and HDP remained as significant ASCVD-REFs, in the multivariable analysis, only the history of HDP (5.35 (1.22–23.38)) and GDM/macrosomia (3.18 (1.05–9.65)) showed independent risks. During the 15-year follow-up, Mets (1.47 (1.05–2.06)) and its components of high waist circumference (1.40 (1.0–1.95)) and high BP (1.52 (1.07–2.15)) significantly increased the risk. These ASCVD-REFs did not improve discrimination or predictive ability.

**Conclusions:**

In a decade follow-up, only conditions specific for women and in longer follow-up, the presence of Mets perse, and its components of high WC and high BP were shown as significant ASCVD-REFs.

## Background

Atherosclerotic cardiovascular disease (ASCVD) is one of the common non-communicable diseases (NCD) with high disability-adjusted life year (DALY) in the Middle East and North Africa (MENA) region [1, 2]. According to the Global Burden of Disease (GBD) estimates, lipid disorders are one of the main causes of morbidity and mortality in this region. DALYs due to high low-density lipoprotein cholesterol (LDL-C) have decreased by 46% during 1990–2017 in Iran, but it is still higher than the global estimate. This could be due to poor control of dyslipidemia despite increased medical therapy [1]. According to the last national study conducted in 2016 among the Iranian population aged over 25 years, more than 80% showed at least one lipid abnormality; among which the prevalence of high non-HDL-C (high-density lipoprotein cholesterol) and hypercholesterolemia was reported as 40% and 27%, respectively [3].

There are several guidelines for lipid management. One of which is the guideline of the 2013 American College of Cardiology/American Heart Association (ACC/AHA) [4], which was validated for the Iranian population [5]. This guideline recommended a risk score that was calculated using pooled cohort equations among individuals without CVD (ASCVD-PCE) [4, 6, 7]. Based on the individuals’ ASCVD-PCE risk score, non-diabetic individuals with LDL-C ≥ 70–189 mg/dl are then classified into low (< 5%), borderline (5–7.5%), intermediate (7.5-20%), and high-risk (≥ 20%). The guideline for primary prevention was updated in 2018/2019 (2018/2019 ACC/AHA) and recommends statin therapy for individuals in the high-risk group and those with borderline and intermediate risk, in case of having atherosclerotic cardiovascular disease risk enhancing factors (ASCVD-REFs) [8] (Table 1), although the U.S Preventive Services Task Force (USPSTF) concluded: “current evidence is insufficient to assess the balance of benefits and harms of adding nontraditional risk factors to existing CVD risk assessment models” [9].

**Table 1:** Recommended ASCVD risk enhancing factors for individuals with borderline and intermediate-risk

Family history of premature CVD
Persistently elevated LDL-C ≥ 160 mg/dl
Chronic kidney disease
Metabolic syndrome
Condition-specific to women (e.g. HDP, premature menopause, GDM/macrosomia)
Inflammatory disease (especially rheumatoid arthritis, psoriasis, HIV)
Ethnicity (e.g. south Asian ancestry)
Lipid biomarkers
Persistently elevated triglycerides ≥ 175 mg/dl
In selected individuals, if measured
hs-CRP ≥ 2 mg/dl
LP (a) levels ≥ 50 mg/dl
apoB ≥ 130 mg/dl
Ankle-brachial index (ABI) < 0.9

*CVD* cardiovascular disease, *LDL-C* low-density lipoprotein cholesterol, *hs-CRP* highly sensitive C-reactive protein; *LP (a)* lipoprotein (a), *GDM* gestational diabetes, *HDP* hypertensive disorders pregnancy

The association between each ASCVD-REFs and CVD has been mentioned in several previously published studies among the general population [6, 10–16]. However, to the best of our knowledge, no studies examined the role of the ASCVD-REFs on hard CVD events (a composite of death from CVD, non-fatal myocardial infarction, or non-fatal stroke) among individuals with borderline and intermediate CVD risk. In the current study, we aimed to examine the predictability of each potential ASCVD-REFs on incident all CVD and hard CVD in non-diabetic individuals, with ASCVD-PCE risk 5-20% (including borderline and intermediate-risk) during 10 and 15-year follow-up. Moreover, we evaluated the added value of these ASCVD-REFs on the predictive power of the ASCVD -PCE score.

## Materials and methods

### Study population

Tehran Lipid and Glucose Study (TLGS) is a community-based prospective cohort study carried out on an Iranian urban population in Tehran. The study aims to determine the prevalence and incidence of non-communicable diseases and related risk factors among people aged ≥ 3 years and encourage a healthy lifestyle and programs for the prevention of NCD. The study has been done in two phases including the first (1999–2001: n = 15,005) and the second (2001-2005; n = 3550) and is planned to continue for at least 20 years with a three-year interval design. The design and methodology of the TLGS study have been reported elsewhere [17].

**Fig. 1:**
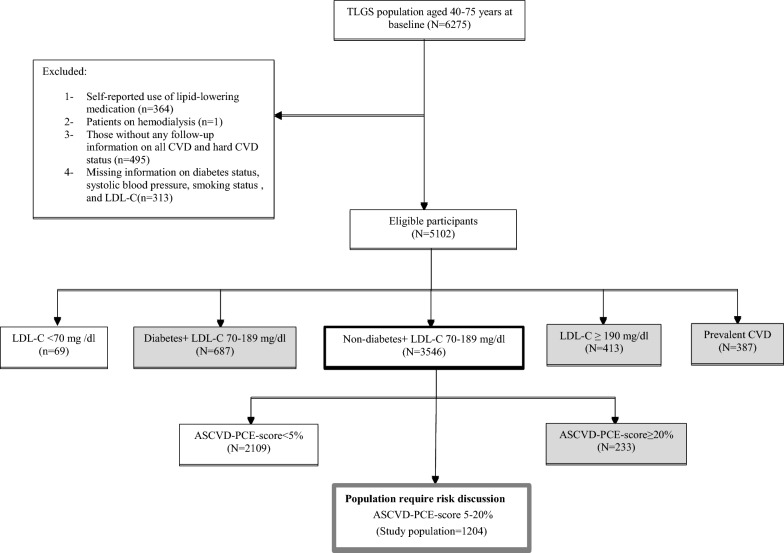
Study population including high risk individuals who are candidate for at least moderate to intensity statin therapy based on 2018/2019 updated ACC/AHA guideline: Tehran lipids and glucose study (1999–2016). Suggested subgroups for statin therapy are highlighted. *CVD* cardiovascular disease, *LDL* low density lipoprotein cholesterol

In the current study, we included 6275 adults aged 40-75 years who entered the first or second phase of the TLGS study. According to the 2013 ACC/AHA guideline, we excluded those with self-reported use of lipid-lowering medication (n = 364) and patients on hemodialysis (n = 1). Other exclusion included missing data for calculating ASCVD-PCE risk score including total cholesterol (TC), HDL-C, triglycerides (TGs), fasting plasma glucose (FPG), 2-hour post-challenge plasma glucose (2 h-PCG), systolic blood pressure (SBP), smoking status (n = 313) as well as those without any information for follow-up on all CVD and hard CVD status (n = 495); leaving us with 5102 individuals. From this number, we excluded those with prevalent CVD (n = 387), LDL-C < 70 mg/dl (n = 69) or ≥ 190 mg/dl (n = 413) and those with type 2 diabetes (T2DM) (n = 687). Finally, of a total of 3546 non-diabetic individuals with LDL-C range 70-189 mg/dl, after excluding individuals with ASCVD-PCE score < 5% (n = 2109) and ≥ 20% (n = 233), 1204 individuals (332 women) were eligible for the analysis of the further risk discussion on the primary prevention of all CVD and hard CVD during 10-year (up to 20 Mach 2010) and 15-year follow-up (up to 20 Mach 2016) (Fig. 1).

This study was approved by the Institutional Review Board (IRB) of the Research Institute for Endocrine Sciences (RIES), Shahid Beheshti University of Medical Sciences, Tehran, Iran, and all participants provided written informed consent.

### Clinical and laboratory measurements

Study participants were interviewed and a standard questionnaire was used to collect demographic information and subjects were questioned about their family history of CVD, premature menopause, hypertensive disorders of pregnancy (HDP), Gestational diabetes mellitus (GDM), macrosomia, and smoking habits. Weight and height were measured to the nearest 100 g while wearing light clothing and with shoes removed. Height was measured in a standing position, using a tape measure, while shoulders were in normal alignment. Waist circumference (WC) was measured with light clothing at the level of the umbilicus. Systolic blood pressure and diastolic blood pressure (DBP) were measured on the right arm after a 15-min rest in a sitting position. The mean of two measurements was considered as the subject’s blood pressure.

Biochemical measurements including FPG, 2 h-PCG, TC, TGs, HDL-C, and creatinine were taken following a 12–14 h overnight fasting from all study participants between 7:00 and 9:00 AM. More details have been described elsewhere [17]. The modified Friedewald formula was used to calculate the LDL-C [18, 19].

### Variable definition

Body mass index (BMI) was calculated as weight (kg) divided by height (m^2^). A positive family history of premature CVD for the study participant was considered as having previously diagnosed CVD in first-degree male aged < 55 and female < 65 year’s relatives. The current smoker was defined as who smokes cigarettes/pipe daily or occasionally. Chronic kidney disease (CKD) was defined as an Estimated Glomerular Filtration Rate (eGFR) of < 60 mL/min per 1.73 m^2^ for longer than 3 months [20]. Type 2 Diabetes (T2DM) was defined if FPG was ≥ 126 mg/dl and/or 2-h PCG was ≥ 200 mg/dl or in case of using anti-diabetic medications. Applying the Joint Interim Statement [21], those who met at least three of the following five criteria were considered to have metabolic syndrome (Mets): [1] WC ≥ 95 cm for both sexes [22]; [2] TGs ≥ 150 mg/dL or lipid-lowering medications [3] HDL-C < 40 mg/dL in males, < 50 mg/dL in females or lipid-lowering medications [4] SBP/DBP ≥ 130/85 mmHg or antihypertensive medication, and [5] FPG ≥ 100 mg/dL or using anti-diabetic medications. GDM/Macrosomia was defined as the self-reported history of GDM or history of having a macrocosmic baby (> 4 kg) [23]. HDP were defined as either preeclampsia or gestational hypertension as reported by participants. Definitions of risk enhancers are presented in Table 1.

### Outcomes

Cardiovascular outcomes details have been published elsewhere [24]. In the TLGS study, each participant is followed-up for any medical event leading to hospitalization during the previous year by telephone call. They were questioned for any medical conditions by a trained nurse and later, a trained physician collected complementary data regarding that event during a home visit and by the acquisition of data from medical files. If required, the outcome assessment committee consisting of an internist, endocrinologist, cardiologist, epidemiologist, and other experts evaluated the collected data to assign a specific outcome for every event. In the current study, all CVD events were defined as a composite measure of any cases of definite and probable myocardial infarction (MI), unstable angina, angiographic proven chronic heart disease (CHD), CHD death, definite or possible stroke, transient ischemic attack or cerebrovascular death. Hard CVD was defined as a composite of death from CVD, non-fatal myocardial infarction, or non-fatal stroke.

### ACC/AHA guideline

The 2013 ACC/AHA guideline was used to calculate the 10-year risk of hard CVD for adults aged 40-75 years based on age, gender, race, SBP, DBP, treatment for hypertension, total cholesterol, HDL-C, T2DM, and current smoking status. Detail for risk equation was reported elsewhere [5, 25]. The 10-year ASCVD-PCE risk score in non-diabetic individuals with LDL-C 70-189 mg/dl was classified into four risk groups; low: < 5%, borderline: 5% to < 7.5%, intermediate: ≥ 7.5% to < 20%, and high-risk: ≥ 20%. According to this guideline moderate to high-intensity statin therapy is recommended for individuals with prevalent CVD, those with LDL-C ≥ 190 mg/dl, diabetic patients, and non-diabetic individuals with LDL-C 70-189 mg/dl with a risk score ≥ of 5%. [26]. This guideline was updated in 2018/2019 and for non-diabetic participants at borderline or intermediate risk (5% to 20%), the presence of ASCVD-REFs (including a family history of premature CVD, CKD, elevated triglycerides (TGs ≥ 175 mg/dl), elevated LDL-C (LDL-C ≥ 160 mg/dl), metabolic syndrome (Mets), and women-specific conditions including premature menopause, GDM/macrosomia, and HDP) may favor initiation of moderate to intensity statin therapy [26]. In the current study to reach full statistical power, we combined borderline and intermediate-risk groups as a single group.

### Statistical analysis

Baseline characteristics of the study population were expressed as mean (95% confidence Intervals: CI) values for continuous variables, and as frequencies (%) for categorical variables. A comparison of the baseline characteristics of the study participants across two risk categories (borderline and intermediate) was done using the t-test for normally distributed continuous variables, the Chi squared test for categorical variables, and the Mann–Whitney test for skewed variables. Cox proportional hazards models were used to evaluating the associations of each risk enhancer including a positive family history of premature CVD, elevated LDL-C ≥ 160 mg/dl, elevated TGs ≥ 175 mg/dl, individuals with CKD, Mets, and conditions specific to women (e.g. HDP and premature menopause) with the incidence of all CVD and hard CVD. We also examined the role of different components of Mets and GDM/macrosomia as another potential ASCVD-REFs.

The event date was defined as the date of the incident all CVD and hard CVD. Those who met the following criteria were censored: leaving the residential area, non-CVD related death, loss to follow-up, or end of follow-up. Cox proportional hazard regression models were used to calculate hazard ratios and 95% confidence intervals (CI) for each recommended ASCVD-REFs. To do the statistical analysis in a hierarchical manner, the role of different REFs was examined both in a univariable (unadjusted) and then a more complex multivariable Cox regression, adjusted for ASCVD-PCE risk score on the incident CVD and hard CVD during 10 and 15-year follow-up. To evaluate the discriminative ability of the model (for CVD and hard CVD separately for 10- and 15-year follow-up), Harrell´s concordance statistic (C-index) were calculated. The z-score test was applied to compare the C-indices of ASCVD-PCE before and after adjustment for significant ASCVD-REFs [27]. For significant risk enhancers specific to women, the C-index of the ASCVD-PCE risk was estimated only for women, on the other hand for other risk enhancers this value was calculated for the whole population. Then, to evaluate the usefulness of each significant ASCVD-REFs the Integrated discrimination improvement (IDI) was used as measures of predictive ability for incident all CVD and hard CVD [28]. The bootstrapping method with 1000 replications was used to report bias-corrected 95% CI [28, 29]. All analyses were conducted using STATA version 12 SE (StataCorp, TX, USA), and a two-tailed p ≤ 0.05 was considered significant.

## Results

The study population consisted of 1204 individuals at baseline with a mean (95% CI) age of 57.5 years (57.0–58.0). Baseline characteristics of the study population according to 10-year ASCVD risk categories are shown in Table 2. The prevalence of Mets, elevated TGs, elevated LDL-C, CKD, and positive family history of premature CVD among the total population and history of conditions specific to women including HDP, premature menopause, and GDM/Macrosomia were 48.5%, 44.5%, 24.7%, 27.7%, 13.3%, 2.1%, 3.0%, and 9.34% respectively. No significant differences were observed between the borderline-risk and intermediate-risk groups considering the prevalence of ASCVD-REFs.

**Table 2:** Baseline characteristics of the study participants according to the 10-year ASCVD risk categories: Tehran lipid and glucose study 1999–2016)

	Total (n = 1204)	10-year ASCVD risk categories		
		Borderline risk (n = 401)	Intermediate risk (n = 803)	p
**10-year ACC/AHA risk-related variables**				
Female gender, n (%)	332 (27.57)	152 (37.9)	180 (22.4)	
Age, years	57.5 (57.0–58.0)	53.9 (53.2–54.7)	59.3 (58.7–59.8)	0.07
SBP (mmHg)	128.3 (127.1–129.4)	123.9 (122.1–125.6)	130.5 (129.1–132.0)	0.001
DBP (mmHg)	80.5 (79.8–81.1)	80.0 (78.9–81.1)	80.7 (79.9–81.6)	0.03
TC (mg/dl)	214.6 (212.7–216.6)	214.5 (211.2–217.8)	214.6 (212.2–217.1)	0.42
HDL-C (mg/dl)	39.2 (38.6–39.8)	39.6 (38.5–40.6)	39.1 (38.4–39.8)	0.11
LDL-C (mg/dl)	138.6 (137.1–140.1)	138.0 (135.5–140.5)	138.9 (137.0–140.8)	0.28
FPG (mg/dl)	92.9 (92.3–93.5)	92.9 (91.9–93.9)	92.9 (92.2–93.6)	0.71
2 h-PCPG (mg/dl)	115.0 (113.1–116.8)	113.6 (110.5–116.7)	115.7 (113.4–118.0)	0.49
Current smoker, n (%)	381 (31.64)	106 (26.4)	275 (34.2)	
Anti-hypertensive medication, n (%)	126 (10.5)	46 (11.5)	80 (10.0)	
ASCVD risk enhancers				
METS, n (%)	583 (48.5)	191 (47.7)	392 (48.9)	0.7
Elevated TG, n (%)	536 (44.5)	180 (44.9)	356 (44.3)	0.85
Elevated LDL-C, n (%)	298 (24.7)	91 (22.7)	207 (25.8)	0.24
HDP^a^, n (%)	7 (2.1)	3 (1.97)	4 (2.22)	0.59
CKD, n (%)	333 (27.7)	100 (24.9)	233 (29.0)	0.13
Family history premature CVD, n (%)	160 (13.3)	62 (15.5)	98 (12.2)	0.12
Premature menopause^a^, n (%)	10 (3.0)	5 (3.29)	5 (2.78)	0.26
GDM/Macrosomia^a^, n (%)	31 (9.34)	18 (11.84)	13 (7.22)	0.15
Other variables				
BMI (kg/m^2^)	26.9 (26.6–27.1)	27.2 (26.7–27.6)	26.7 (26.4–27.0)	0.37
WC (cm)	92.3 (91.7–93.0)	92.0 (90.9–93.0)	92.5 (91.8–93.3)	0.85
eGFR (ml/min/1.73 m^2^)	67.4 (91.7–93.0)	68.8 (67.6–70.0)	66.7 (65.9–67.5)	0.59
TG (mg/dl)	164.0 (121.0–235.5)	165.0 (122.0–2.34)	163.0 (120.0–237.0)	0.73
10-year hard CVD event, n (%)	73 (6.06)	16 (4.0)	57 (7.1)	0.03
15-year hard CVD event, n (%)	138 (11.46)	33 (8.23)	105 (13.1)	0.01
10-year all CVD event, n (%)	181 (15.03)	39 (9.73)	142 (17.68)	< 0.001
15-year all CVD event, n (%)	296 (24.6)	70 (17.46)	226 (28.14)	< 0.001

*ACC/AHA* American College of Cardiology and the American Heart Association, *ASCVD* atherosclerotic cardiovascular disease, *BMI* body mass index, *WC* waist circumference, *SBP* systolic blood pressure, *DBP* diastolic blood pressure, *FPG* fasting plasma glucose, *2h-PCPG* 2-hour post-challenge plasma glucose, *CKD* chronic kidney disease, *eGFR* estimated glomerular filtration rate, *CVD* cardiovascular disease, *TC* total cholesterol, *LDL-C* low-density lipoprotein cholesterol, *HDL-C* high-density lipoprotein cholesterol, *TG* triglyceride, *GDM* gestational diabetes, *Mets* metabolic syndrome, Elevated TG: TG ≥ 177.0 mg/dl; Elevated LDL-C: LDL-C ≥ mg/dl; *HDP* hypertensive disorders pregnancy, *p* p-value

Values are shown as Mean (95% CI) and number (%), (for continuous and categorical variables, respectively); for TG values are shown as median (Interquartile range)

^a^Reported only among females

During the 10-year follow-up, the cumulative incidence of all CVD was 181 (15.03%); the corresponding values for the 15-year follow-up were 296 (24.6%). Considering the hard CVD, the cumulative incidence for the whole population was 73 (6.06%) and 138 (11.46%) during the 10- and 15-year follow-up, respectively. Adults in the intermediate-risk category experienced a higher incidence of all CVD and hard CVD compared with borderline risk categories (Table 2).

### Hard CVD results

**Fig. 2:**
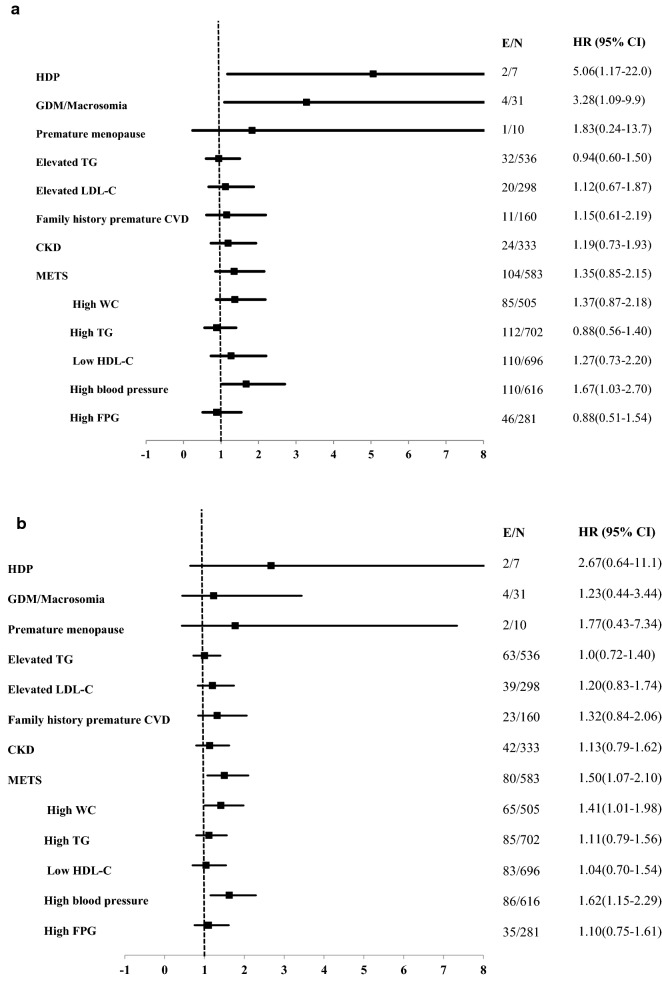
Univariable hazard Ratios (HR) and 95% Confidence Intervals (CI) of ASCVD risk enhancers for borderline/intermediate-risk groups according to the 2019 ACC/AHA guideline on the Primary Prevention of Cardiovascular Disease with incident hard CVD during the median 10-year (**a**) and 15-year (**b**) follow-up: Tehran Lipid and Glucose Study 1999–2016. *ASCVD* atherosclerotic cardiovascular disease, *WC* waist circumference, *FPG* fasting plasma glucose, *CKD* chronic kidney disease, *LDL-C* low-density lipoprotein cholesterol, *HDL-C* high-density lipoprotein cholesterol, *TG* triglyceride, *Mets* metabolic syndrome, *CVD* cardiovascular disease, *GDM* gestational diabetes, *HDP* hypertensive disorders pregnancy. Elevated TG: TG ≥ 175 mg/dl; Elevated LDL-C: LDL-C ≥ 160 mg/dl. E: number of the event; N: number of sample size. *HDP and Premature menopause Reported only among females

Among the population with borderline/intermediate ASCVD risk, the univariable hazard ratios (HRs) with 95% CI of ASCVD-REFs for hard CVD during 10 and 15-year follow-up are shown in Fig. 2a, b, respectively. For a decade follow-up, GDM/macrosomia (3.28 (1.09–9.9)), and the history of HDP (5.06 (1.17–22.0)) among women and high BP component of Mets (1.67 (1.03–2.70)) in total population remained as significant ASCVD-REFs. In the multivariable analysis adjusted for ASCVD-PCE score (Table 3), only the history of HDP (5.35 (1.22–23.38)) and GDM/macrosomia (3.18 (1.05–9.65))among women showed significant risks, whereas, in the ASCVD-PCE adjusted score, high BP component of Mets showed a non-significant association (1.53 (0.94–2.48); p = 0.08) during 10 years of follow-up. As shown in Table 3, the C-statistics of discrimination for the model with and without significant risk enhancers were the same for incident hard CVD. According to the IDI results, we observed that these ASCVD-REFs did not improve the predictive power of the ASCVD-PCE risk score. The maximum relative IDI of the mentioned risk enhancers was 0.6% for the prediction of hard CVD (Table 3).

**Table 3:** Additional predictive power for Hard CVD by the ASCVD risk enhancers^a^: Tehran lipid and glucose study (1999–2016)

	ACC/AHA model HR (95%)	p	Enhanced model HR (95%)		p
**Median 10-year follow-up**					
Model components^b^ 1					
ASCVD-PCE Score	0.96 (0.83–1.10)	0.53	0.95 (0.82–1.10)		0.46
HDP			*5.35 (1.22*–*23.38)*		*0.026*
Model predictive performance indexes 1					
C-index^c^ (95% CI)	0.52 (0.42–0.61)	< 0.001	0.56 (0.49–0.63)		< 0.001
IDI (95% CIs)			0.006 (− 0.03–0.04)		0.74
Model components^b^ 2					
ASCVD-PCE Score	0.96 (0.83–1.10)	0.53	0.96 (0.84–1.11)		0.63
GDM/Macrosomia			3.18 (1.05–9.65)		0.041
Model predictive performance indexes 2					
C-index^c^ (95% CI)	0.52 (0.42–0.61)	< 0.001	0.60 (0.48–0.74)		< 0.001
IDI (95% CIs)			0.0007 (− 0.03–0.03)		0.97
Model components 3					
ASCVD-PCE Score	*1.09 (1.03*–*1.14)*	*0.001*	*1.08 (1.02*–*1.14)*		*0.004*
High blood pressure			1.53 (0.94–2.48)		0.08
Model predictive performance indexes 3					
C-index^c^ (95% CI)	0.60 (0.58–0.62)	< 0.001	0.62 (0.49–0.74)		< 0.001
IDI (95% CIs)			0.001 (− 0.002–0.006)		0.55
**Median 15-year follow-up**					
Model components 1					
ASCVD-PCE Score	*1.06 (1.03*–*1.10)*	*0.001*	*1.06 (1.025*–*1.11)*		*0.001*
METS			*1.47 (1.05*–*2.06)*		*0.026*
Model predictive performance indexes 1					
C-index^c^ (95% CI)	0.58 (0.55–0.62)	< 0.001	0.58 (0.51–0.65)	< 0.001	
IDI (95% CIs)			0.003 (− 0.003–0.01)	0.36	
Model components 2					
ASCVD-PCE Score	*1.06 (1.03*–*1.10)*	*0.001*	*1.06 (1.02*–*1.10)*	*0.001*	
High WC			*1.40 (1.0*–*1.95)*	*0.05*	
Model predictive performance indexes 2					
C-index^c^ (95% CI)	0.58 (0.55–0.62)	< 0.001	0.58 (0.51–0.65)	< 0.001	
IDI (95% CIs)			0.002 (− 0.003–0.007)	0.46	
Model components 3					
ASCVD-PCE Score	*1.06 (1.03*–*1.10)*	*0.001*	*1.06 (1.02*–*1.10)*	*0.004*	
High blood pressure			*1.52 (1.07*–*2.15)*	*0.02*	
Model predictive performance indexes 3					
C-index^c^ (95% CI)	0.58 (0.55–0.62)	< 0.001	0.58 (0.51–0.65)	< 0.001	
IDI (95% CIs)			0.002 (− 0.004–00.008)	0.45	

*ACC/AHA* American College of Cardiology and the American Heart Association, *ASCVD* atherosclerotic cardiovascular disease, *Mets* metabolic syndrome, *GDM* gestational diabetes, *HDP* hypertensive disorders pregnancy, *CVD* cardiovascular disease, *AUC* area under the curve, *IDI* integrated discrimination improvement, *CI* confidence interval, *p* p-value

^a^Significant ASCVD risk factors

^b^Only among women

^c^C-index: Concordance index. The difference between C-indices was not significant

^d^C-index: indicate the discriminative ability of the model

During the 15-year follow-up, Mets (1.47 (1.05–2.06)) and its components of high waist circumference (1.40 (1.0–1.95)) and high BP (1.52 (1.07–2.15)) significantly increased the risk of hard CVD in multivariable analysis. However, these ASCVD-REFs. did not improve the predictive power of the ASCVD-PCE risk score (Table 3).

### All CVD events

**Fig. 3:**
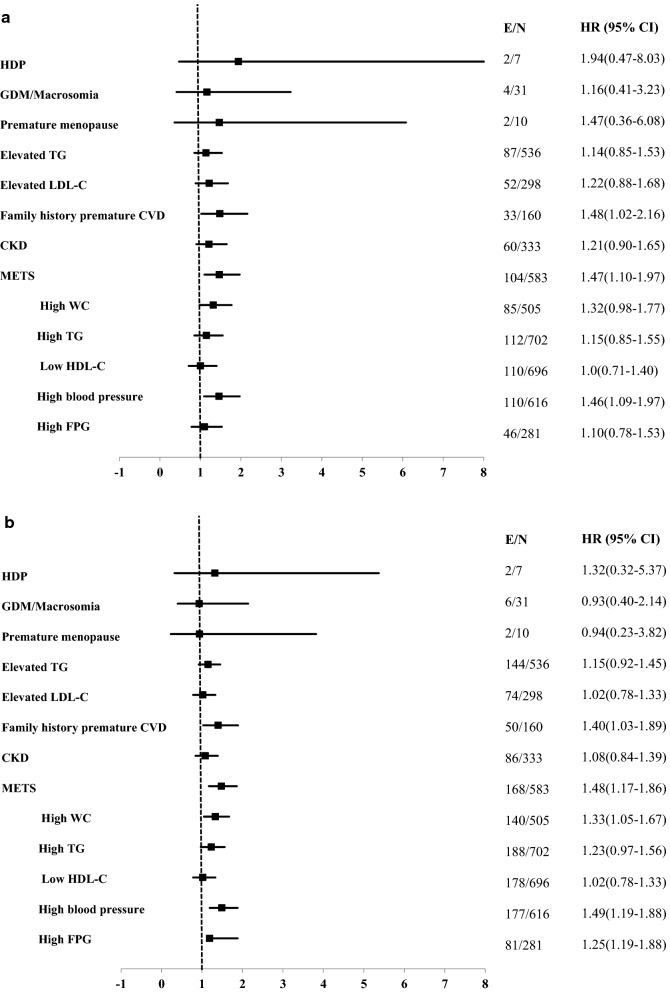
Univariable hazard Ratios (HR) and 95% Confidence Intervals (CI) of ASCVD risk enhancers for borderline/intermediate-risk groups according to the 2019 ACC/AHA guideline on the Primary Prevention of Cardiovascular Disease with Incident all-CVD during the median 10-year (**a**) and 15-year (**b**) follow-up: Tehran Lipid and Glucose Study 1999–2016. *ASCVD* atherosclerotic cardiovascular disease, *WC* waist circumference, *FPG* fasting plasma glucose, *CKD* chronic kidney disease, *LDL-C* low-density lipoprotein cholesterol, *HDL-C* high-density lipoprotein cholesterol, *TG* triglyceride, *Mets* metabolic syndrome, *CVD* cardiovascular disease, *GDM* gestational diabetes, *HDP* hypertensive disorders pregnancy. Elevated TG: TG ≥ 175 mg/dl; Elevated LDL-C: LDL-C ≥ 160 mg/dl. E: number of the event; N: number of sample size. *HDP and Premature menopause Reported only among females

For all CVD events, during the 10-year follow-up, family history premature CVD (1.48 (1.02–2.16)), Mets (1.47 (1.10–1.97)) and its high BP component (1.46 (1.09–1.97)) significantly increased the risk in univariable analysis (Fig. 3a). In the multivariable analysis with ASCVD-PCE adjusted score, family history premature CVD (1.54 (1.05–2.24)), Mets (1.45 (1.08–1.95)), and high BP component of Mets (1.34 (1.0–1.82)) showed significant risks for incident all CVD during 10 years of follow-up. The C-statistics of discrimination for the model with and without significant risk enhancers were the same for incident all CVD and these ASCVD-REFs did not improve the predictive power of the ASCVD-PCE risk score. The maximum relative IDI of the aforementioned risk enhancers was 0.2% for the prediction of all CVD events (Table 4).

**Table 4:** Additional predictive power for all CVD by the ASCVD risk enhancers^a^: Tehran lipid and glucose study (1999–2016)

	Median 10-year follow-up				Median 15-year follow-up			
	ACC/AHA model HR (95%)	p	Enhanced model HR (95%)	p	ACC/AHA model HR (95%)	p	Enhanced model HR (95%)	p
Model components 1								
ASCVD-PCE Score	*1.08 (1.05*–*1.12)*	< *0.001*	*1.08 (1.05*–*1.12)*	< *0.001*	*1.07 (1.04*–*1.10)*	< *0.001*	*1.07 (1.04*–*1.10)*	< *0.001*
Family history premature CVD			*1.54 (1.05*–*2.24)*	*0.025*			*1.42 (1.05*–*1.93)*	*0.02*
Model predictive performance indexes 1								
C-index^b^ (95% CI)	0.6 (0.56–0.63)	< 0.001	0.6 (0.54–0.66)	< 0.001	0.59 (0.57–0.62)	< 0.001	0.59 (0.55–0.63)	< 0.001
IDI (95% CIs)			0.002 (− 0.005–0.01)	0.56			0.001 (− 0.002–0.03)	0.57
Model components 2								
ASCVD-PCE Score	*1.08 (1.05*–*1.12)*	< *0.001*	*1.08 (1.05*–*1.12)*	< *0.001*	*1.07 (1.04*–*1.10)*	< *0.001*	*1.07 (1.04*–*1.10)*	< *0.001*
METS			*1.45 (1.08*–*1.95)*	*0.013*			*1.45 (1.15*–*1.82)*	*0.002*
Model predictive performance indexes 2								
C-index^b^ (95% CI)	0.6 (0.56–0.63)	< 0.001	0.6 (0.56–0.64)	< 0.001	0.59 (0.57–0.62)	< 0.001	0.59 (0.57–0.62)	< 0.001
IDI (95% CIs)			0.001 (− 0.005–0.008)	0.73			0.002 (− 0.004–0.008)	0.55
Model components 3								
ASCVD- PCE Score	*1.08 (1.05*–*1.12)*	< *0.001*	*1.08 (1.05*–*1.12)*	< *0.001*	*1.07 (1.04*–*1.10)*	< *0.001*	*1.07 (1.04*–*1.10)*	< *0.001*
High WC			1.30 (0.97–1.75)	0.08			*1.32 (1.05*–*1.66)*	*0.02*
Model predictive performance indexes 3								
C-index^b^ (95% CI)	0.6 (0.56–0.63)	< 0.001	0.6 (0.53–0.66)	< 0.001	0.59 (0.57–0.62)	< 0.001	0.59 (0.56–0.62)	< 0.001
IDI (95% CIs)			0.002 (− 0.003–0.007)	0.48			0.002 (− 0.003–0.007)	0.5
Model components 4								
ASCVD-PCE Score	*1.08 (1.05*–*1.12)*	< *0.001*	*1.08 (1.04*–*1.11)*	< *0.001*	*1.07 (1.04*–*1.10)*	< *0.001*	*1.06 (1.04*–*1.09)*	< *0.001*
High blood pressure			*1.34 (1.0*–*1.82)*	*0.05*			*1.38 (1.09*–*1.75)*	*0.007*
Model predictive performance indexes 4								
C-index^b^ (95% CI)	0.6 (0.56–0.63)	< 0.001	0.6 (0.55–0.64)	< 0.001	0.59 (0.57–0.62)	< 0.001	0.59 (0.58–0.60)	< 0.001
IDI (95% CIs)			0.002 (− 0.004–0.007)	0.57			0.002 (− 0.0.008)	0.41

*ACC/AHA* American College of Cardiology and the American Heart Association, *ASCVD* atherosclerotic cardiovascular disease, *Mets* metabolic syndrome, *GDM* gestational diabetes, *HDP* hypertensive disorders pregnancy, *CVD* cardiovascular disease, *AUC* area under the curve, *IDI* integrated discrimination improvement, *CI* confidence interval, *p* p-value

^a^Significant ASCVD risk factors

^b^C-index: Concordance index. The difference between C-indices was not significant

The univariable HRs (95% CI) of ASCVD-REFs for a 15 years follow-up showed that family history premature CVD 1.40 (1.03–1.89), Mets (1.48 (1.17–1.86)) and its components including high WC (1.33 (1.05–1.67)) and high BP (1.49 (1.19–1.88)) increased the risk incident CVD (Fig. 3b). The C-statistics of discrimination for the model with and without significant risk enhancers were the same for incident all CVD and these ASCVD-REFs did not improve the predictive power of the ASCVD-PCE risk score events for 15-year follow-up. (Table 4).

As for sensitivity analysis, when we replaced the BP cut off of 130/80 mmHg, the ACC/AHA definition of hypertension [30], with 130/85 mmHg, the results remained essentially unchanged (data not are shown).

## Discussion

In the current study conducted among a large population in the MENA region, we examined for the first time, the impact of ASCVD-REFs among participants with borderline/intermediate ASCVD-PCE score on incident all CVD and hard CVD events during more than a decade follow-up. As for hard CVD, in a multivariable analysis adjusted for ASCVD-PCE risk score, HDP and GDM/macrosomia showed a signal of significant risk during a 10-year follow-up while Mets, its high BP (i.e. BP > 130/85) and high WC components remained significant ASCVD-REFs during the 15-year follow-up. For all CVD events, in multivariable analysis, a family history of premature CVD and Mets were the remaining ASCVD-REFs during both follow-up periods. Importantly among Mets components, high BP as another potential ASCVD-REF was also predictive of all CVD. Moreover, abdominal obesity remained a significant ASCVD-REFs for all CVD only during the 15-year follow-up. However, none of the above mentioned significant ASCVD-REFs had an added value on ASCVD-PCE score in the prediction of the CVD events.

### Women-specific conditions

In the multivariable analysis of our study, HDP remained significant ASCVD-REFs for hard CVD during a 10-year follow-up. In line with our results, a recent review has shown that women with HDP have about twofold risk for the development of CVD [31]. Moreover, a study by Young L et al. emphasized an increased risk of CVD and CVD mortality in women with a history of preeclampsia according to meta-analysis [32]. Recently, in a meta-analysis pooling results of 9 cohort studies, researchers found that gestational hypertension and preeclampsia were associated with 67% and 73% higher risk of CVD events; however, the heterogeneity between included studies was high [33]. However, as in our study adding HDP to the already existing ASCVD-PCE score did not or slightly improve discrimination or reclassification [31].

GDM/macrosomia was a significant potential ASCVD-REFs when adjusted for the risk score (HR = 3.18 (1.05–9.65)). The recent meta-analysis using data of 8 cohort studies was demonstrated the odds ratio of subsequent CVD in women with GDM was about 70% higher compared to women without GDM [33]. Another recent meta-analysis was also reported that women with GDM had a twofold greater risk for future CVD events; however, after restricting the population to women who did not develop T2DM during the follow-up the related risk of incident CVD was attenuated but remained significant (relative risk 1.56 (95% CI 1.04–2.32)) [34]. Although the development of diabetes after GDM might be a large contributing factor for incident CVD other pathophysiology have certainly important roles [32]. It is thought that women with a history of dysglycemia have an underlying cardio-metabolic phenotype that makes them susceptible to GDM and CVD. Glucose screening during pregnancy could identify women at risk for CVD [35].

The exact pathophysiology of the harmful impact of HDP and GDM/Macrosomia remains unclear, but it was generally shown that it was related to placental and/or vascular dysfunction [33]. The following mechanisms might be underling this pathophysiology such as endothelial dysfunction, vasoconstriction, and vascular resistance due to several causes including increased inflammatory and immunologic factors, reduced nitric oxide, and reactive oxygen species released from the ischemic and dysfunctional placental and mitochondrial dysfunction [12].

It would be beneficial to recommend statin therapy to women with intermediate/borderline risk who have a history of GDM and/or pre-eclampsia with a shared decision-making process and application of personalized medicine to reduce the incidence of CVD among those with a risk of 5–20%, considering the high burden and lower appropriate diagnosis of CVD among women, globally and in the MENA region [36].

### Family history of CVD

Focusing on family history of CVD, a meta-analysis on 26 studies, showed a pooled estimate of 1.31 (95% CI 1.17–1.47; I^2^ 58%) for the paternal history of CVD and 1.48 (95% CI 1.30–1.68; I^2^ 45%) for the maternal history of CVD, for incident CVD [37]. In our study, this risk factor was associated with a significant incidence of all but not hard CVD over 10-year and 15-year follow-up, indicating its independent genetic role in the occurrence of CVD events, although its presence did not reclassify the population at borderline/intermediate risk.

### Metabolic syndrome and its components

Mets did not remain significant ASCVD-REFs for hard CVD during the 10-year follow-up, but it remained significant for hard CVD during the 15-year follow-up and all CVD during both follow-up periods in our population. A meta-analysis of 87 studies showed that Mets is associated with a twofold increase in the risk of CVD, CV mortality, MI, and stroke, and a 1.5 fold increase in the risk of all-cause mortality [38]. Among the Iranian population, we also showed that during 10 years of follow-up, the presence of Mets, independent of traditional risk factors, was associated with 97% and 120% increased risk of all CVD events in men and women respectively [39].

Among different Mets components, we showed high BP had an independent and consistent role in the occurrence of cardiovascular events, although its presence did not reclassify the study population in borderline/intermediate risk category. In a recent pooled cohort study consisting of 82,717 US adults, it was shown that elevated BP was the Mets component most consistently present in Mets combinations that were significantly and most strongly associated with mortality. The authors also found that BP ≥ 130/85 mmHg in the absence of other risk factors was significantly associated with mortality in both genders [40]. This strong association between high BP and risk of CVD and hard CVD events in our study could be due to the high burden of high blood pressure in the MENA region in the background of the unfavorable trend of obesity, physical inactivity, Westernized diet as well as other psycho-socio-economic factors [1, 41]. The 2017 guideline for the high BP of the ACC/AHA [30], updated the 2003 Seventh Report of the Joint National Committee (JNC7) [42] and the 2014 eight-panel member report (JNC8) [43] guideline in terms of the new definition for hypertension, candidates for pharmacotherapy and blood pressure target goals. Accordingly, the 2017 ACC/AHA guideline suggests a lower threshold of SBP/DBP for the definition of hypertension (130/80 mmHg vs. 140/90 mmHg, respectively), compared to the 2003 JNC7. Additionally, the 2017 AHA/ACC guideline-recommended antihypertensive medication at the level of SBP/DBP 130/80 mmHg for the elder population aged ≥ 65 years and those with high cardiovascular risk including cases with prevalent CVD or population with 10-year predicted cardiovascular risk ≥ 10% using PCE; the issues not addressed in previous guidelines. In the pooled cohort equation, the SBP and antihypertensive medication are two factors for calculating the ASCVD-PCE score. However, current study showed that among the population with ASCVD-PCE risk between 5% and 20%, CVD events occur even at moderately elevated BP (i.e. ≥ 130/85 mmHg), further substantiating the importance of identifying and early treatment of these population not only with anti-hypertensive mediation but also with statin therapy.

Among Mets components, central obesity (i.e. high WC) had the second strongest association with hard CVD among borderline/intermediate-risk individuals. A meta-regression analysis among more than 250,000 participants was shown that the presence of abdominal obesity was significantly associated with incident CVD events and the authors suggested that this factor should be incorporated into CVD risk assessments [44].

### Other factors

Improving risk prediction is not easy [45]. C-reactive protein (hs-CRP) and ankle-brachial index (ABI) have great clinical potential as ASCVD-REFs, but their clinical significance in CVD prediction in terms of calibration, discrimination, and reclassification is yet uncertain [46]. Unfortunately, we did not have data on novel atherosclerotic risk factors such as highly sensitive hs-CRP and measures of vascular damage that precede overt clinical CVD (i.e. ABI and coronary artery calcium score (CAC score)). We used a nested case–control study to assess the effect of hs-CRP in the short-term prediction of cardiovascular disease outcomes in the Iranian population. Results showed that when traditional cardiovascular risk factors are known, the measurement of hs-CRP has no additional value on the predictive power of the model [47].

### Strengths and limitations

This study had some strength. Firstly, to the best of our knowledge, this is the first study to evaluate the added value of ASCVD-REFs on the ASCVD-PCE risk score of individuals with borderline/intermediate risk for all CVD and hard CVD events. Secondly, we assessed the risk score for an extended follow-up period of 15 years. Limitations of the study include 1) lack of information about other ASCVD-REFs such as lipoprotein (a), Apo B, viral infections including hepatitis B, C and, human immunodeficiency deficiency virus (HIV) 2) given the limited number of events, we pooled borderline and intermediate-risk as a single group and no sex-stratified analysis was performed, 3) The significant risk of HDP for hard CVD during a 10-year follow-up was not stable considering the limited number of events. Last but not least, the current study was performed in Iran, a country located in MENA, a region adversely affected by political instability, social conflict, and war [1]. Moreover, in Iran, the implementation of economic sanctions during the long periods lead to scarcity of health-care resources and fall of the country’s revenues, devaluation of the national currency, and increase of inflation and unemployment leading to significant psychological stresses [2, 48, 49]. These mentioned factors potentially deteriorate people’s overall welfare and limiting their ability to reach healthy foods and some of the lifesaving medicines. In the current study, we had no data related to the above factors, moreover, the guideline does not consider the potential impact of these important psycho-socio-economic variables.

## Perspective and conclusion

As for future studies, firstly, we recommend further studies to evaluate the association of number, duration, and severity of each of the ASCVD-REFs on the risk of CVD. Secondly, further cohort studies are recommended to assess the association of Mets components as independent ASCVD-REFs with hard CVD. Thirdly, we strongly recommend assessing the consistency of our results in other regions of the country, especially rural areas.

In summary, among Iranian non-diabetic individuals aged 40-75 years old with borderline/intermediate-risk, women with a history of HDP and/or GDM/macrosomia and individuals with Mets, BP ≥ 130/85 mmHg, or central obesity might benefit from informed decision-making regarding initiation or intensification of statin therapy along with lifestyle modification.

## Data Availability

The datasets used and/or analyzed during the current study are available from the corresponding author on reasonable request.

## Abbreviations

ASCVD-REFs: Atherosclerotic cardiovascular disease risk enhancing factors
ASCVD-PCE: Atherosclerotic cardiovascular disease risk enhancing factors using pooled cohort equations
ACC/AHA: American College of Cardiology/American Heart Association
BMI: Body mass index
CI: Confidence intervals
CVD: Cardiovascular disease
DBP: Diastolic blood pressure
eGFR: Estimated glomerular filtration rate
FPG: Fasting plasma glucose
HR: Hazard ratio
HDL-C: High-density lipoprotein cholesterol
2 h-PCG: 2-hour post-challenge plasma glucose
LDL-C: Low-density lipoprotein cholesterol
Mets: Metabolic syndrome
MENA: Middle East and North Africa
NCD: Non-communicable disease
PCE: Pooled cohort equations
SBP: Systolic blood pressure
T2DM: Type 2 diabetes
TLGS: Tehran lipid and glucose study
TC: Total cholesterol
TGs: Total cholesterol, triglycerides
WC: Waist circumference
HDP: Hypertensive disorders of pregnancy
